# Development of a recombinant Newcastle disease virus-vectored vaccine for infectious bronchitis virus variant strains circulating in Egypt

**DOI:** 10.1186/s13567-019-0631-5

**Published:** 2019-02-11

**Authors:** Hassanein H. Abozeid, Anandan Paldurai, Berin P. Varghese, Sunil K. Khattar, Manal A. Afifi, Sahar Zouelfakkar, Ayman H. El-Deeb, Magdy F. El-Kady, Siba K. Samal

**Affiliations:** 1 Virginia-Maryland Regional College of Veterinary Medicine, University of Maryland, College Park, MD USA; 2 Faculty of Veterinary Medicine, Cairo University, Giza, Egypt; 3 Faculty of Veterinary Medicine, Beni-Suef University, Beni-Suef, Egypt

## Abstract

Infectious bronchitis virus (IBV) causes a major disease problem for the poultry industry worldwide. The currently used live-attenuated vaccines have the tendency to mutate and/or recombine with circulating field strains resulting in the emergence of vaccine-derived variant viruses. In order to circumvent these issues, and to develop a vaccine that is more relevant to Egypt and its neighboring countries, a recombinant avirulent Newcastle disease virus (rNDV) strain LaSota was constructed to express the codon-optimized S glycoprotein of the Egyptian IBV variant strain IBV/Ck/EG/CU/4/2014 belonging to GI-23 lineage, that is prevalent in Egypt and in the Middle East. A wild type and two modified versions of the IBV S protein were expressed individually by rNDV. A high level of S protein expression was detected in vitro by Western blot and immunofluorescence analyses. All rNDV-vectored IBV vaccine candidates were genetically stable, slightly attenuated and showed growth patterns comparable to that of parental rLaSota virus. Single-dose vaccination of 1-day-old SPF White Leghorn chicks with the rNDVs expressing IBV S protein provided significant protection against clinical disease after IBV challenge but did not show reduction in tracheal viral shedding. Single-dose vaccination also provided complete protection against virulent NDV challenge. However, prime-boost vaccination using rNDV expressing the wild type IBV S protein provided better protection, after IBV challenge, against clinical signs and significantly reduced tracheal viral shedding. These results indicate that the NDV-vectored IBV vaccines are promising bivalent vaccine candidates to control both infectious bronchitis and Newcastle disease in Egypt.

**Electronic supplementary material:**

The online version of this article (10.1186/s13567-019-0631-5) contains supplementary material, which is available to authorized users.

## Introduction

Infectious bronchitis (IB) is an acute, highly contagious viral disease of chickens. IB affects chickens of all ages and based on the organ system affected the disease is manifested in three major clinical forms—respiratory, renal and reproductive. IB causes great economic losses in the poultry industry worldwide [1, 2]. Infectious bronchitis virus (IBV) is a member of the genus *Gammacoronavirus* in the family *Coronaviridae*. The viral genome is a single-stranded, positive-sense RNA of about 27.6 Kb in length [3]. The 5′-two-third of the viral genome codes for the non-structural proteins responsible for RNA replication and transcription. The 3′-one-third of the viral genome codes for four structural proteins, namely, spike (S), envelope (E), membrane (M) and nucleocapsid (N) proteins, in addition to several non-structural proteins [3].

The S protein of IBV is heavily glycosylated and plays a major role in eliciting protective immune responses. It is present as trimers on the surface of the virion and contains conformation dependent epitopes [4]. The S protein is cleaved post-translationally by host cell proteases into S1 (N-terminal, globular head domain) and S2 (C-terminal, stalk domain) subunits [5–7]. The S1 subunit is the most variable subunit that harbors the major neutralizing epitopes [8, 9] and the receptor binding domain (RBD) responsible for viral attachment and tissue tropism [5]. The S2 subunit is the most conserved subunit and is involved in mediating membrane fusion and viral entry [5]. The S2 protein also contains minor neutralizing epitopes and contributes to the avidity of S1 protein [10]. It has been shown that a tyrosine motif located in the cytoplasmic tail of the S protein is responsible for the intracellular retention [11]. This tyrosine residue is essential for productive virus infection [12]. However, the role of this tyrosine residue on induction of protective immune response by IBV S protein has not been studied.

Many different IBV serotypes and genotypes circulate worldwide [2, 6, 13]. These serotypes arise due to high frequency of mutations and/or recombination events [14, 15]. Cross-protection between different serotypes is variable or poor [16].

In Egypt, classical and variant strains of IBV co-circulate causing frequent disease outbreaks [17, 18]. The majority of IBV variant strains reported in Egypt belong to GI-23 lineage [19]. The Egyptian variant strains are mostly related to strains IS/885/00 and IS/1494/06, also known as Israeli Variant II [17, 18]. Israeli Variant II-related serotypes have also been reported in many countries in the Middle East and North Africa [20] and recently, in Poland [21] and Turkey [22].

Live-attenuated vaccines have been highly successful in controlling IB in the field [23, 24]. However, live-attenuated vaccines provide cross-protection against some of the IBV variants but not all [24–26]. Furthermore, use of live-attenuated IBV vaccines can lead to production of variant IBV strains by mutations and/or recombination [27, 28].

In Egypt, classical and variant live-attenuated vaccine strains, mainly H120 and 793B, are used to control IB. Although these vaccines provide good protection against some of the variant viruses, they provide poor protection against the prevalent variant field viruses belonging to GI-23 lineage. These live-attenuated vaccines are also a source for generation of new variant viruses [27, 29, 30]. Therefore, an alternative vaccine strategy will be beneficial to control IB outbreaks in Egypt.

Viral vectored vaccines provide an alternative approach to live-attenuated IBV vaccines. A vectored vaccine expressing the protective antigen of IBV will not lead to creation of variant viruses. Attempts have been made to express S1 or S2 subunits of S protein using fowl pox virus, herpes virus and adenovirus vectors [31–34]. However, none of these vaccines are effective in providing complete protection against IBV, suggesting the need for new vaccine vectors.

Use of Newcastle disease virus (NDV) as a vaccine vector for IBV holds great promise. NDV is a member of the genus *Avulavirus* in the family *Paramyxoviridae* [35]. NDV causes a highly contagious disease with substantial mortality in chickens [36]. The natural avirulent NDV strain LaSota is widely used as a live NDV vaccine in chickens for more than 60 years with a good record of safety and stability. NDV replicates efficiently in the respiratory tract of chickens inducing mucosal immunity at the site of IBV entry and it also elicits strong humoral and cell-mediated immune responses crucial for clearance of IBV [37]. Moreover, it can be used as a dual vaccine against IBV and NDV.

Recombinant NDV (rNDV) has been used previously as a vaccine vector to evaluate the protective efficacy of S1, S2 and S proteins of IBV [38–40]. It was reported that rNDV expressing the S2 protein provided only partial protection against virulent IBV challenge [39]. rNDV expressing the IBV S1 protein provided partial protection after a single vaccination and better protection was observed after a booster vaccination [38]. Recently, it was shown that the rNDV expressing the whole S protein of classical IBV M41 strain provided better protection than the rNDVs expressing S1 or S2 protein of IBV [40].

The goal of this study was to evaluate the protective efficacies of three forms of the S protein of Egyptian IBV GI-23 lineage variant strain IBV/Ck/EG/CU/4/2014 using NDV as a vaccine vector. In addition to the expression of wild type S protein, a second S protein was expressed in which the tyrosine residue in the cytoplasmic tail was mutated to alanine, and the modified S protein was fused to the last 12 amino acids of NDF F protein. A third S protein was expressed in which only the cytoplasmic tail (CT) of S protein was replaced with last 12 amino acids of NDV F protein CT. The CT modification was done to enhance incorporation of IBV S protein into NDV envelope. The protective efficacies of the three IBV S proteins were evaluated by homologous challenge with the Egyptian strain IBV/Ck/EG/CU/4/2014.

## Materials and methods

### Cells and viruses

Human epidermoid carcinoma (HEp-2) and chicken embryo fibroblast (DF-1) cell lines were cultured in Dulbecco’s minimal essential medium (DMEM) with 10% fetal bovine serum. Specific pathogen free (SPF) embryonated chicken eggs (ECE) were obtained from Charles River Laboratories, Manassas, VA, USA. The Egyptian IBV strain IBV/Ck/EG/CU/4/2014 belonging to GI-23 lineage (GenBank accession number: KY805846) [27], NDV strain LaSota, virulent NDV strain Texas GB (GenBank accession number: GU978777.1) and modified vaccinia strain Ankara expressing T7 polymerase (MVA-T7), a kind gift from Dr Bernard Moss, were used in this study. All work related to virulent NDV was performed in our USDA certified enhanced animal biosafety level 3 (ABSL-3+) facility.

### Construction of recombinant NDV-vectored IBV vaccine candidates

**Figure 1:**
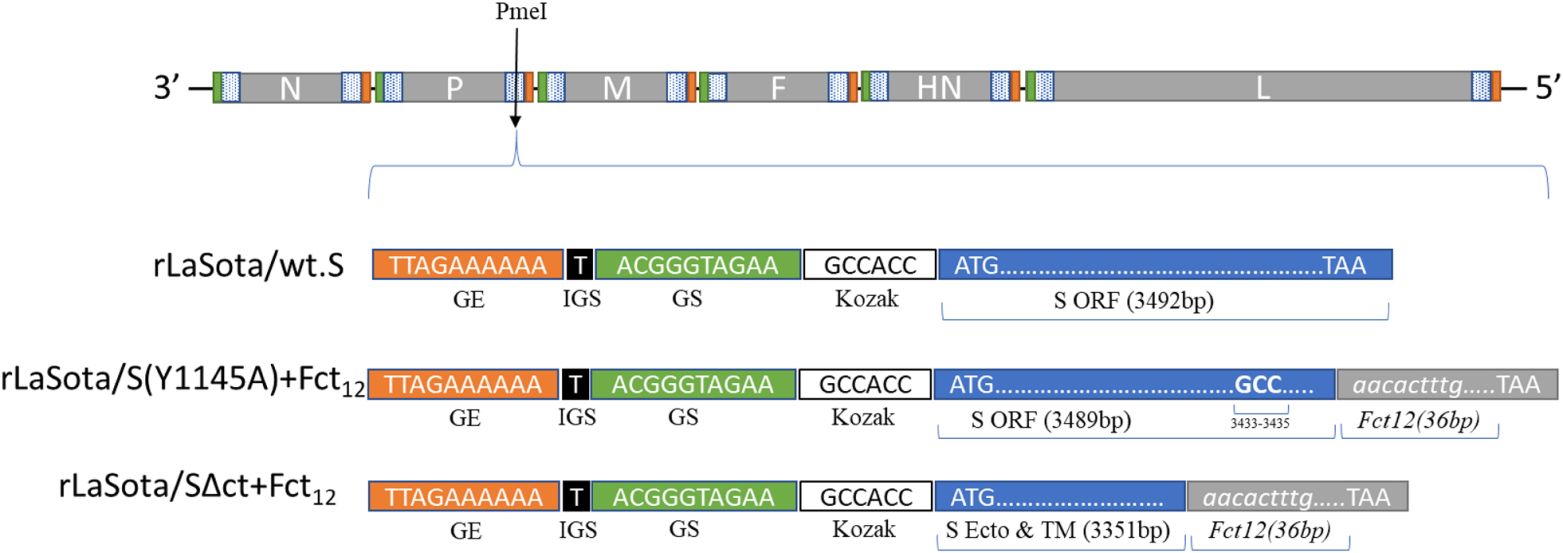
**Construction of three recombinant NDVs containing different forms of the S gene of Egyptian IBV variant strain EG/CU/4/2014.** Schematic representation of different forms of chicken-codon-optimized IBV S gene into NDV strain LaSota antigenome cDNA using *PmeI* site between P and M genes to generate IBV vaccine candidates. In each transcriptional cassette, NDV gene-end (GE), intergenic sequence (IGS), gene-start (GS) and Kozak sequences were added upstream of the IBV S ORF. In rLaSota/wt.S, the full sequence of the IBV S gene without any modification (Blue box) was inserted. In rLaSota/S(Y1145A) + Fct_12_, the tyrosine residue present at position 1145 of the IBV S protein was changed into alanine (GCC nt 3453–3455) and the modified S gene was fused to the last 12 amino acids of NDV fusion protein cytoplasmic tail domain (Grey box). In rLaSota/SΔct + Fct_12_, ecto- and transmembrane domains of IBV S protein were fused with the last 12 amino acids of NDV fusion protein cytoplasmic tail domain.

Three different forms of chicken-codon-optimized S gene of the Egyptian IBV strain IBV/Ck/EG/CU/4/2014 were inserted individually between phosphoprotein (P) and matrix (M) protein genes in the LaSota antigenomic cDNA backbone using *PmeI* site [41] to generate three different recombinant NDV vectored IBV vaccine candidates (Figure 1). In the first recombinant, rLaSota/wt.S, the full chicken-codon-optimized sequence of the IBV S gene without any modification was inserted. In the second recombinant, rLaSota/S(Y1145A) + Fct_12_, the tyrosine residue present at position 1145 of the IBV S protein was mutated to Alanine (Y1145A) by PCR mutagenesis and the modified S gene was fused to the last 12 amino acids of NDV fusion (F) protein cytoplasmic domain using overlap PCR. In the third recombinant, rLaSota/SΔct + Fct_12_, the cytoplasmic tail of the IBV S gene was replaced by the last 12 amino acids of NDV fusion protein using fusion PCR technique. All the three forms of the IBV S gene were inserted as an independent transcription cassette flanked by the gene-start (GS) and gene-end (GE) sequences of the NDV and the “rule of six” was ensured in the final length of the recombinant NDV genome for efficient replication [42]. Briefly, the codon-optimized IBV S gene—preceded with GE, intergenic sequence (IGS), GS and Kozak sequences, and flanked by *PmeI* sites—was synthesized and cloned in pUC57 (pUC57-CO.S) by GenScript^®^ (Additional file 1A). To construct rLaSota/wt.S, pUC57-CO.S was digested with *PmeI* (NEB), and the released insert was gel purified and ligated into full-length rLaSota clone which was predigested by *PmeI* and dephosphorylated by thermostable shrimp alkaline phosphatase (TSAP) (Promega). To construct rLaSota/S(Y1145A) + Fct_12_, primer sets (1 and 2) and (3 and 4) were used to induce the “Y1145A-mutation” and add the last 12 aa of NDV F protein using pUC57-CO.S as template (Table 1). The two PCR products were then fused using primers (1 and 4) by fusion PCR. The fused PCR product was then digested by *SmaI* and *StuI* (NEB), and ligated into pUC57-CO.S which was predigested by *SmaI* and by *StuI*, its sequence is naturally present downstream to the S gene, to create pUC57-CO.S(Y1145A) + Fct_12_ (Additional file 1B). To construct rLaSota/SΔct + Fct_12_, primer set (1 and 5) was used to replace the cytoplasmic tail of IBV S protein by the last 12 aa of NDV F protein using pUC57-CO.S as template. The PCR product was then digested by *SmaI* and *StuI* and ligated into pUC57-CO.S which is predigested by *SmaI* and *StuI* to create pUC57-CO.SΔct + Fct_12_ (Additional file 1C). The newly formed pUC57-CO.S(Y1145A) + Fct_12_ and pUC57-CO.SΔct + Fct_12_ were then digested with *PmeI* and the released inserts were ligated individually into full-length rLaSota clone which was predigested by *PmeI* and dephosphorylated by TSAP to construct rLaSota/S(Y1145A) + Fct_12_ and rLaSota/SΔct + Fct_12_, respectively. The sequences of all inserts were confirmed by DNA nucleotide sequencing. The recombinant viruses were rescued using the reverse genetic technique as previously described [43]. Rescued recombinant viruses were plaque purified and propagated in 10-day-old SPF ECE. The infective allantoic fluids were harvested, aliquoted and stored at −70 °C for further work. The insertion of the different forms of IBV S genes were detected by RT-PCR using LaSota specific primers flanking the *PmeI* site and confirmed by nucleotide sequencing.

**Table 1:** **Primers used for construction of the rNDV expressing IBV S protein vaccine candidates**

Primer number	Primer name	Primer sequence
1	COS-2167F (*SmaI*)^a^	ACTCTGAAAGACCTGATCTGCG
2	COS-(YA) Rev	AGTTGT**GGC**GTAGGAGCTTTTC^b^
3	COS-(YA) Fwd	AGCTCCTAC**GCC**ACAACTTTTG^c^
4	COS-R (*StuI*-*PmeI*-Fct_12_)	AGGCCT*GTTTAAAC*TCACATTTTAGTGGTTGCCCTCATTTGATCCAAAGTGTT**CACTGATTTCTTGGGTCTGTAC**^d^
5	COSΔct-R (*StuI*-*PmeI*-Fct_12_)	AGGCCT*GTTTAAAC*TCACATTTTAGTGGTTGCCCTCATTTGATCCAAAGTGTT**AAAGAAGATCCACCCCAGGATC**^e^

^a^The primer sequence is just upstream to the naturally present *SmaI* sequence at the nt position 2167 of the codon-optimized S gene sequence.

^b^The sequence “GGC” in bold indicates the reverse complementary mutagenic sequence of alanine residue at nt position 3433–3435 of the codon-optimized S gene sequence.

^c^The sequence “GCC” in bold indicates the mutagenic sequence of alanine residue at nt position 3433–3435 of the codon-optimized S gene sequence.

^d^Underlined sequence indicates *StuI*, *italicized* sequence in indicates *PmeI*, sequence in regular font indicates nt coding for optimized last 12 aa of NDV F protein, sequence in bold indicates the N terminus of IBV S specific sequence.

^e^Underlined sequence indicates *StuI*, *italicized* sequence indicates *PmeI*, sequence in regular font indicates nt coding for optimized last 12 aa of NDV F protein, sequence in bold indicates the N terminus of the transmembrane domain of IBV S specific sequence.

### In vitro characterization of the NDV-vectored IBV vaccine candidates

To compare the multicycle growth kinetics of rLaSota/wt.S, rLaSota/S(Y1145A) + Fct_12_ and rLaSota/SΔct + Fct_12_ with that of parental rLaSota, DF-1 cells cultured in six-well plates were infected by the recombinant viruses at MOI of 0.01. After 1 h of virus adsorption, cells were washed twice with serum-free DMEM and then incubated with DMEM containing 2% FBS and 10% normal allantoic fluid in 5% CO_2_ incubator at 37 °C. Supernatant media (200 µL) were collected from each well at 12 h intervals for 72 h and replaced by equal volume of fresh DMEM with 2% FBS and 10% allantoic fluid each time. Virus titers of the collected supernatant samples were determined by tissue culture infective dose 50% (TCID_50_) titration assay. Briefly, dilutions from 10^−1^ to 10^−8^ of each sample were inoculated on to DF-1 cell monolayers in 96-well plates. Four replicate wells were used for each dilution. After 1 h of adsorption, cell monolayers were overlaid with DMEM containing 2% FBS and 10% normal allantoic fluid and incubated in 5% CO_2_ incubator at 37 °C for 6 days. The TCID_50_ was calculated according to Reed and Muench method [44]. To check the genetic stability, the three recombinant viruses were passaged 5 times in ECE and the presence of the different forms of the S gene was then checked by RT-PCR using OneTaq^®^ One-Step RT-PCR Kit (New England Biolabs^®^, Inc) and DNA sequencing using BigDye^®^ Terminator v3.1 cycle sequencing kit (Applied Biosystems, USA) in ABI 3130×l genetic analyzer (Applied Biosystems).

Surface and intracellular expression of the different forms of the S gene were examined in infected DF-1 cells by immunofluorescence and by Western blot analyses. Briefly, DF-1 cells grown to 90% confluency on glass slide chambers (Nunc Lab-Tek™ II, ThermoFisher, USA) were infected with each virus at MOI 0.1. Two chambers were used for each virus. At 24 hours post-infection (hpi), cells were fixed with 2% paraformaldehyde solution for 15 min and then quenched by 125 mM glycine for 5 min. Cells were then permeabilized with 0.1% Triton X-100 for 10 min. To demonstrate the surface expression, the cell permeabilization step was excluded. Then, cells were blocked with 5% goat serum for 30 min and then incubated with chicken polyclonal anti-IBV antibody generated against the Egyptian strain IBV/Ck/EG/CU/4/2014 (diluted 1:100) for 2 h, and with goat anti-chicken fluorescein isothiocyanate (FITC) conjugated antibody (diluted 1:500) for 45 min. All these steps were carried out at room temperature. Cell nuclei were stained with 4′,6-diamidino-2-phenylindole (DAPI) and cells were visualized under the Confocal Zeiss LSM 510 fluorescence microscope.

For Western blot analysis, infected DF-1 cells were lysed in radioimmunoprecipitation assay (RIPA) buffer and incubated on ice for 10 min then clarified by centrifugation at maximum speed at 4 °C. The supernatant was mixed with SDS–PAGE sample buffer [125 mM Tris–HCl (pH 6.8), 4.6% SDS, 50 mM DTT, 0.005% bromophenol blue and 20% glycerol], denatured by heating in boiling water bath for 5 min, resolved by SDS–PAGE and analyzed by Western blot using chicken polyclonal anti-IBV antibody.

To determine the incorporation of the S protein of IBV into the NDV particles, recombinant viruses were partially purified by ultracentrifugation. Briefly, 10 mL of infective allantoic fluid of each recombinant virus was passed through 30% sucrose cushion for 2 h at 28 000 rpm at 4 °C and the virus pellet was resuspended in 500 µL PBS. Partially purified LaSota virus was used as a negative control. The resuspended pellet was then mixed with SDS-PAGE sample buffer, heat denatured, resolved by SDS-PAGE, and analyzed by Western blot using chicken polyclonal anti-IBV antibody.

The pathogenicity of each recombinant NDV-vectored IBV vaccine candidate virus was compared to that of parental LaSota virus by the mean death time (MDT) assay in 10-day-old SPF ECE [45]. Briefly, 10-fold serial dilutions of the infective allantoic fluid were made in sterile PBS, and 100 µL of the dilutions ranging from 10^−6^ to 10^−9^ were inoculated into four 10-day-old embryonated eggs via allantoic sac route. Eggs were incubated at 37 °C and examined three times daily for 7 days to record times of embryo mortality. The MDT was determined as mean time (h) for the minimum lethal dose of virus to kill all the inoculated embryos.

### Evaluation of the protective efficacy of single-dose vaccination against IBV and virulent NDV

To evaluate the protective efficacy against IBV, sixty 1-day-old SPF White Leghorn chickens were divided into 5 groups and housed in negative pressure isolators with feed and water provided ad libitum. Four groups containing 15, 10, 15 and 10 birds were vaccinated with 10^6^ PFU/100 µL of rLaSota, rLaSota/wt.S, rLaSota/S(Y1145A) + Fct_12_ and rLaSota/SΔct + Fct_12_, respectively, via oculonasal route. A fifth group, containing 10 birds, served as unvaccinated control.

Three weeks post-immunization, blood samples were collected from all birds to determine the level of hemagglutination inhibition (HI) antibody titers in the serum against NDV [46] and the antibody response against IBV using ELISA. After blood collection, 10 birds from all vaccinated groups and 5 birds from the unvaccinated control group were challenged with 10^4.2^ EID_50_ of IBV/Ck/EG/CU/4/2014 via oculonasal route. Birds were observed and scored for clinical signs until 10 days post-challenge (dpc) before they were euthanized using CO_2_ euthanasia procedure approved by IACUC, University of Maryland. Scores include, 0 for normal, 1 for mild ocular discharge and mild nasal discharge and/or sneezing, 2 for heavy ocular discharge and heavy nasal discharge and/or coughing, and 3 for tracheal rales and/or mouth breathing. At 5 dpc, tracheal and oropharyngeal swabs were collected from all birds to detect the level of viral shedding based on IBV-specific iTaq™ one-step universal probe RT-qPCR kit [47].

To evaluate the protective efficacy against NDV, 5 birds from groups vaccinated with rLaSota and rLaSota/S(Y1145A) + Fct_12_ in addition to the unvaccinated control group were challenged with 100 chicken lethal dose 50% (CLD_50_) of virulent NDV strain Texas GB, via oculonasal route, in the ABSL-3 + facility. The NDV strain Texas GB is used as a NDV challenge strain in the US. Birds were observed for clinical signs and mortality until 10 dpc. In IBV and NDV challenge studies, death/mortality was not the endpoint, birds were scored for physical appearance, physical activity, neurological and/or respiratory signs. When reached a cumulative score of 6, birds were euthanized using CO_2_ euthanasia procedure approved by IACUC, University of Maryland. Euthanized birds were scored as dead on the same day.

### Evaluation of the protective efficacy of a prime-boost vaccination strategy against IBV

Fifty 1-day-old SPF White Leghorn chicks were divided into 5 groups of 10 birds each and housed in negative pressure isolators with feed and water provided ad libitum. Birds in groups 1, 2, 3 and 4 were immunized (primed) with 10^6^ PFU of rLaSota, rLaSota/wt.S, rLaSota/S(Y1145A) + Fct_12_ and rLaSota/SΔct + Fct_12_, respectively, at day 1 of age and boosted at day 14 of age via oculonasal route. Birds in group number 5 served as unvaccinated control. On day 35 of age (3 weeks post-boost), birds in all groups were challenged with 10^5.2^ of IBV/Ck/EG/CU/4/2014 via ocular route. Clinical signs were recorded daily for 10 dpc. At 5 dpc, tracheal and oropharyngeal swabs were collected from each bird to determine the level of viral shedding using IBV-specific iTaq one-step universal probe RT-qPCR kit [47]. Blood samples were collected at day 35 of age (just before challenge) to determine the serum HI antibody titers against NDV and antibody response against IBV using ELISA.

### Determination of the antibody response against IBV

IBV-specific immune response was determined using ELISA. Briefly, ELISA plates (Nunc Maxisorp) were coated with heat-inactivated purified IBV/Ck/EG/CU/4/2014 in carbonate-bicarbonate buffer, pH 9.8, at 4 °C overnight. The IBV-coated plates were then blocked by 5% skim milk powder in PBS overnight at 4 °C. Plates were then loaded with serum samples diluted 1:100 in 5% skim milk for 2 h at room temperature. Plates were washed, and IBV-specific antibodies were detected using the reagents in the commercial IBV ELISA kit (Synbiotics, USA) according to the manufacturer instructions. Absorbance (optical density) was measured at 405 nm using BIOTEK ELx808 ELISA reader.

### Statistical analysis

Statistical significance between different groups were determined using one-way analysis of variance (ANOVA) followed by Tukey’s multiple comparison test using Prism 7 (GraphPad Software Inc., San Diego, CA) with *P* value < 0.05.

## Results

### Construction of rNDVs and analysis of IBV S protein expression

**Figure 2:**
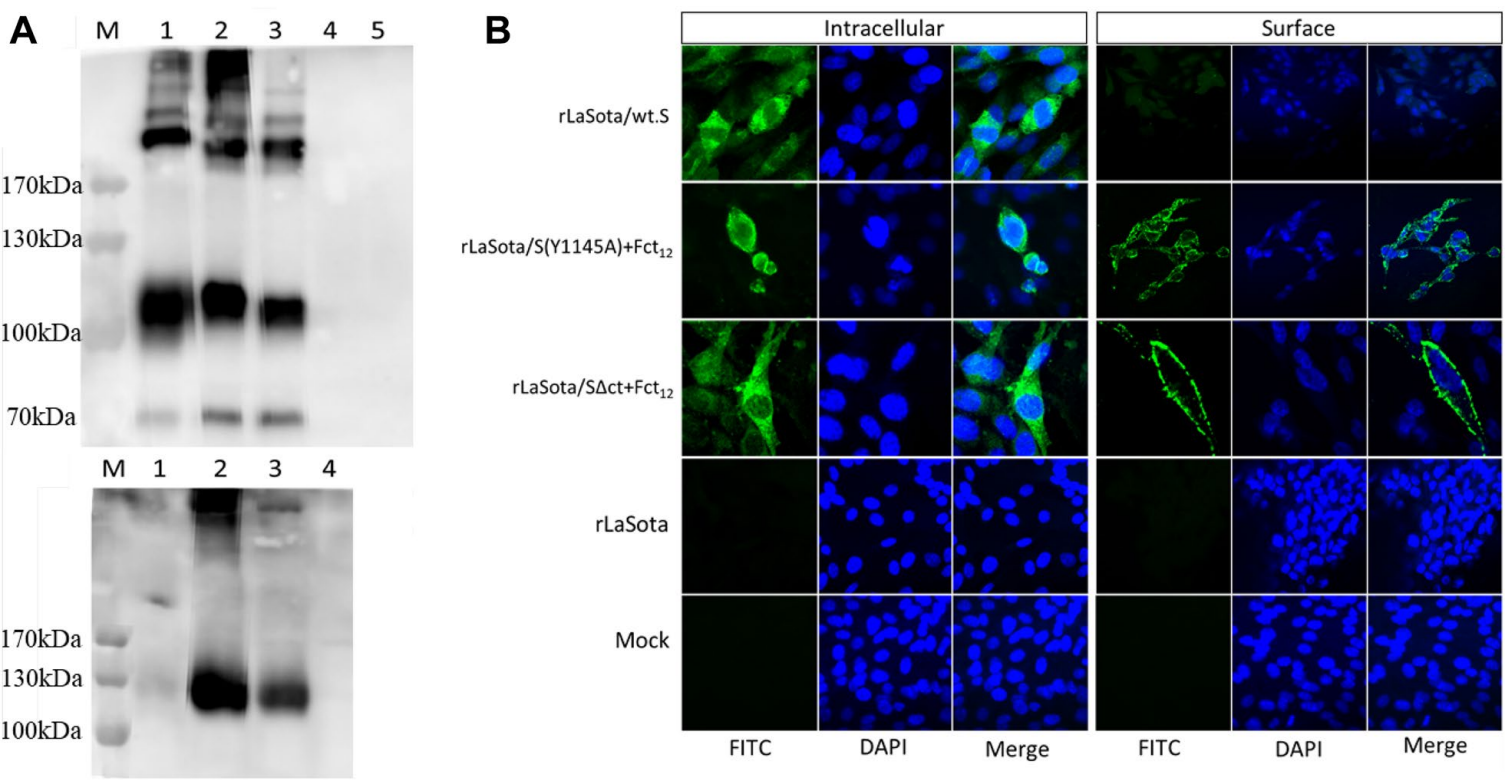
**Expression of IBV S protein in DF-1 cells infected with the rNDV-vectored IBV vaccine candidates. A** Western blot analysis of IBV S protein. DF-1 cell lysates were analyzed 48 hpi by Western blot analysis using polyclonal IBV antisera (top panel). Analysis of incorporation of IBV S protein into purified rNDV virions from infective allantoic fluid by Western blot analysis using polyclonal IBV antisera (bottom panel). M, Marker; 1, rLaSota/wt.S; 2, rLaSota/S(Y1145A) + Fct_12_; 3, rLaSota/SΔct + Fct_12_; 4, rLaSota; 5, mock-infected. **B** Immunofluorescence analysis of the intracellular and surface expression of IBV S protein in DF-1 cells infected with the rNDV-vectored IBV vaccine candidates. The cells were probed with polyclonal IBV antisera followed by detection with FITC-conjugated goat anti-chicken IgG antibodies (green) and DAPI (blue) and subsequently visualized using a confocal Zeiss LSM 510 fluorescence microscope.

All the three forms of IBV S gene were inserted into the full-length cDNA clone of NDV strain LaSota between the P and M genes, and their corresponding recombinant viruses, rLaSota/wt.S, rLaSota/S(Y1145A) + Fct_12_ and rLaSota/SΔct + Fct_12_, were successfully rescued. The RT-PCR and DNA sequencing confirmed the presence of the three different forms of the IBV S gene in the rLaSota genomes. After the fifth passage in ECE, the presence of IBV S genes were detected by RT-PCR in the three recombinant viruses and confirmed by DNA sequencing, indicating that all recombinant viruses were genetically stable. The expression of S protein by rNDVs was detected by Western blot. Western blot analysis of the infected DF-1 cell lysates revealed three bands indicating S, S1 and S2 proteins of IBV. However, no bands were detected in mock and in rLaSota-infected DF-1 cells (Figure 2A, top panel). The expression of S protein by rNDVs was further confirmed by immunofluorescence. Immunofluorescence analysis showed only intracellular expression of IBV S protein in DF-1 cells infected with rLaSota/wt.S; whereas, both intracellular and surface expression of IBV S protein were observed in cells infected with rLaSota/S(Y1145A) + Fct_12_ and rLaSota/SΔct + Fct_12_. No expression of IBV S protein was detected in mock and rLaSota-infected cells (Figure 2B).

To determine whether the S protein expressed by rNDVs was incorporated into NDV virion, the rNDVs collected from infected eggs were partially purified through sucrose cushion. Western blot analysis of the partially purified recombinant viruses showed expression of the IBV S protein in rLaSota/S(Y1145A) + Fct_12_ and rLaSota/SΔct + Fct_12_ but not in rLaSota/wt.S or in rLaSota. This indicates that fusion with the last 12 amino acids of NDV fusion protein resulted in the incorporation of IBV S protein into the NDV virion (Figure 2A, bottom panel).

### In vitro characterization of IBV vaccine candidates

**Figure 3:**
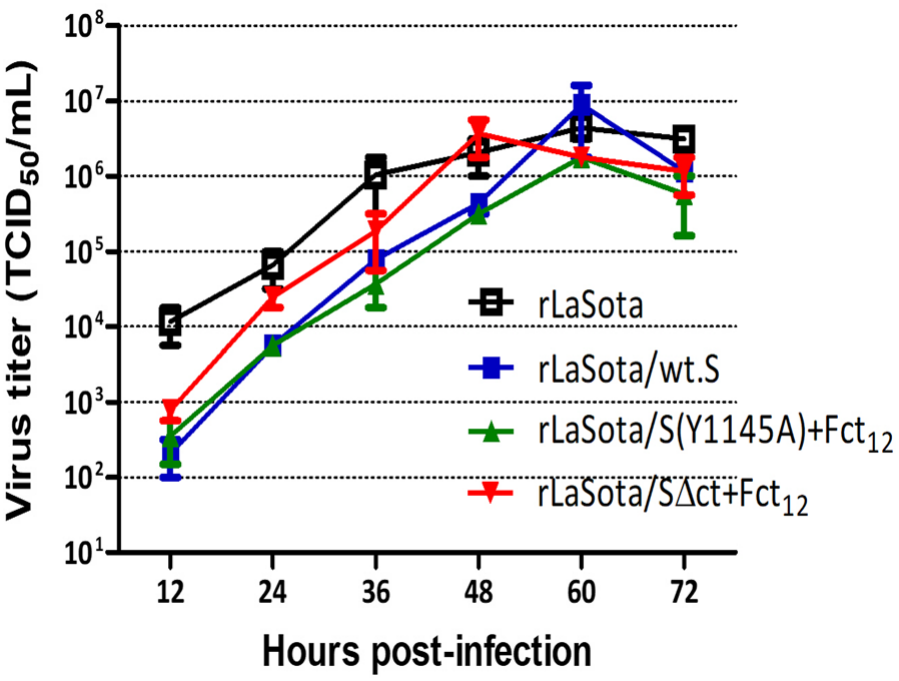
**Growth kinetics of rLaSota and rLaSota expressing different forms of IBV S protein of Egyptian IBV variant strain EG/CU/4/2014.** DF-1 cells were infected with the indicated viruses at MOI of 0.01. Normal fresh chicken embryo allantoic fluid was added to the medium as exogenous source of protease. Samples of the supernatant media were collected at 12 h intervals and the viral titers were determined by limiting dilution on DF-1 cells. Means and SEM of two independent experiments are plotted at each time point.

The multicycle growth kinetics of the rLaSota/wt.S, rLaSota/S(Y1145A) + Fct_12_ and rLaSota/SΔct + Fct_12_ were evaluated in DF-1 cells in the presence of exogenous protease. The growth of all three recombinants were comparable with that of the parental rLaSota. The growth of rLaSota/wt.S, rLaSota/S(Y1145A) + Fct_12_ and rLaSota/SΔct + Fct_12_ were slightly lower than parental rLaSota till 48 hpi but by 60 hpi they reached titers similar to parental rLaSota. rLaSota, rLaSota/wt.S and rLaSota/S(Y1145A) + Fct_12_ reached maximum titers of 10^6.6^, 10^6.9^ and 10^6.3^ TCID_50_/mL, respectively, at 60 hpi, and rLaSota/SΔct + Fct_12_ reached a maximum titer of 10^6.6^ TCID_50_/mL at 48 hpi (Figure 3).

The pathogenicity of each rNDV was evaluated in ECE by MDT assay. rLaSota/wt.S, rLaSota/S(Y1145A) + Fct_12_ and rLaSota/SΔct + Fct_12_ showed MDT values of 134 h, 137 h and 139 h, respectively, which were higher than that of the parental rLaSota (98 h) indicating that they are slightly attenuated than the parental rLaSota.

### Protective efficacy of rNDVs expressing IBV S protein after single-dose vaccination

**Figure 4:**
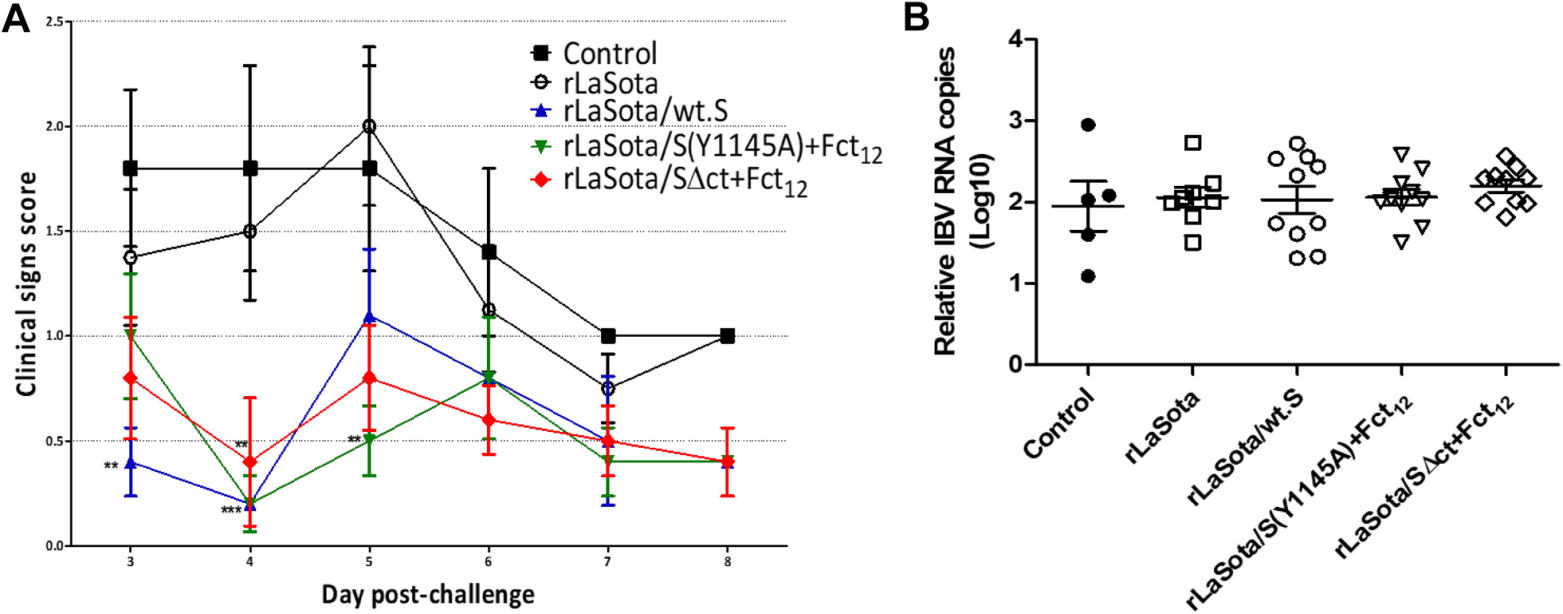
**Protective efficacy of single-dose vaccination of rNDV-vectored IBV vaccine candidates in 1-day-old SPF chickens after IBV challenge. A** Clinical signs in chickens immunized with the indicated vaccine candidates and challenged with 10^4.2^ EID_50_ of Egyptian IBV variant strain EG/CU/4/2014. The birds were monitored daily for 10 days for clinical signs scoring (0 = normal, 1 = mild ocular discharge, mild nasal discharge and/or sneezing 2 = heavy ocular discharge, heavy nasal discharge and/or coughing 3 = tracheal rales and/or mouth breathing). Mean score and SEM are plotted for each group. Statistically significant differences versus the control group at each time point are indicated by asterisk. **B** Determination of viral load by RT-qPCR quantitative analysis of IBV RNA in tracheal and oropharyngeal swabs collected at 5 days post IBV challenge. Each point indicates the viral load for an individual chicken, the lines indicate the mean viral load for each group, and the error bars indicate SEM.

To evaluate the protective efficacy of rNDVs expressing IBV S protein, 1-day-old SPF chicks were immunized with single-dose of each virus and challenged after 3 weeks with the Egyptian strain IBV/Ck/EG/CU/4/2014. Birds in all groups showed respiratory signs of varying severity, including lacrimation, nasal discharge, sneeze, cough, tracheal rales and mouth breathing, starting from 3 dpc. Birds vaccinated with rLaSota/wt.S, rLaSota/S(Y1145A) + Fct_12_ and rLaSota/SΔct + Fct_12_ showed significantly milder signs than birds in the control and rLaSota-vaccinated groups, especially at 4 and 5 dpc (Figure 4A). RT-qPCR results showed no significant reduction in tracheal shedding of IBV between the control and the vaccinated groups (Figure 4B).

**Figure 5:**
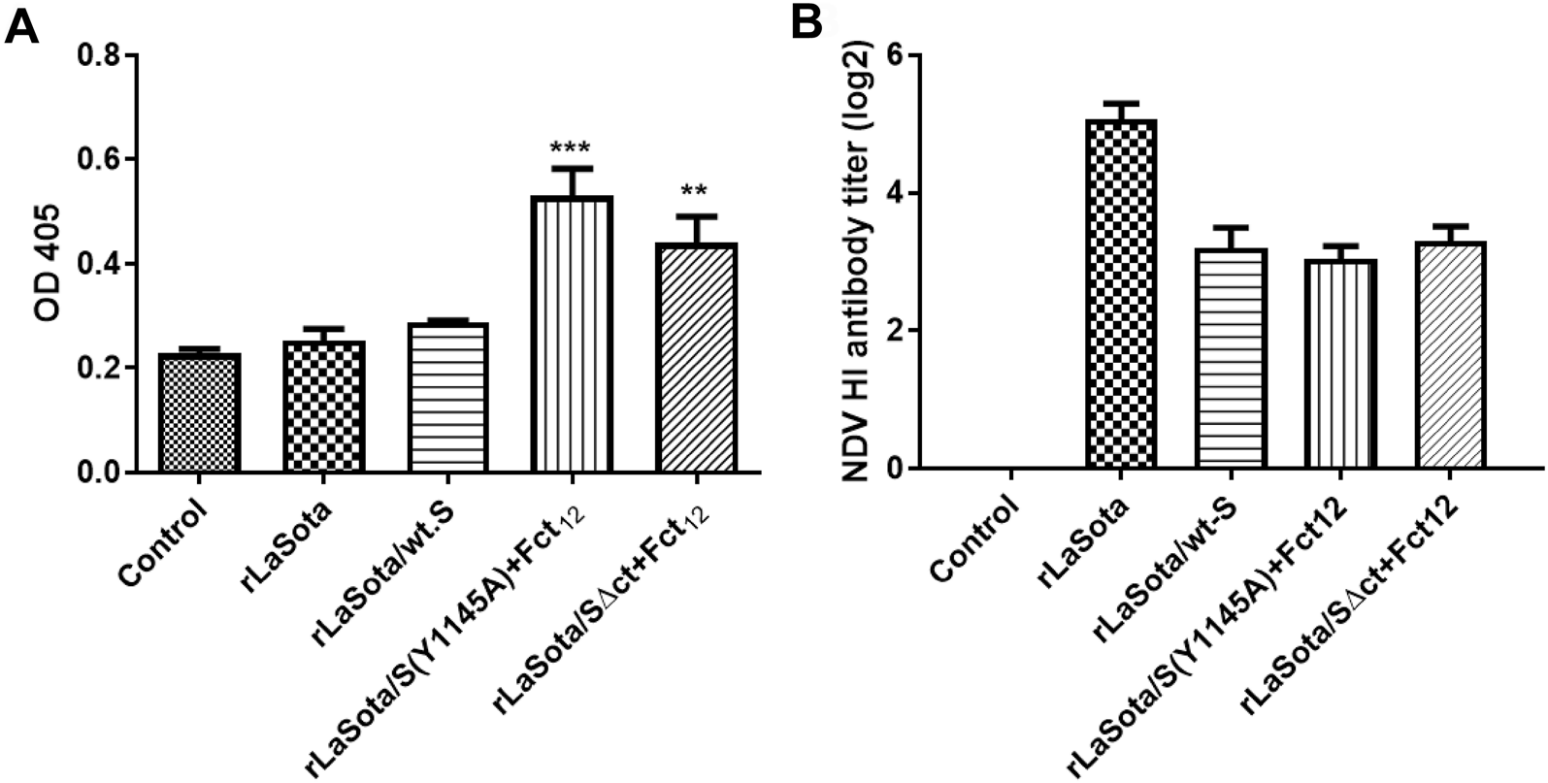
**Antibody responses against IBV (A) and NDV (B) in 1-day-old SPF chicks vaccinated with different rNDV-vectored IBV vaccine candidates. A** The level of IBV-specific antibodies in chicken serum samples on week 3 (prior to challenge) was determined by ELISA. Mean absorbance values and SEM are shown for each group. Statistically significant differences versus the control group are indicated by asterisk. **B** The level of NDV-specific antibody titers in chicken serum samples on week 3 (prior to challenge) was determined by hemagglutination inhibition (HI) assay. Mean and SEM of each group are shown.

The IBV specific antibodies produced in vaccinated birds were assessed by ELISA. At 3 weeks post-immunization, chickens vaccinated with rLaSota/S(Y1145A) + Fct_12_ and rLaSota/SΔct + Fct_12_ showed significant increase in ELISA antibody titers compared to the control non-vaccinated group (*P* < 0.001 and *P *< 0.01, respectively). However, the chickens vaccinated with rLaSota and rLaSota/wt.S did not show IBV specific ELISA antibody titers (Figure 5A).

The protective efficacy of rNDVs expressing IBV S protein was evaluated against virulent strain of NDV. Upon challenge with velogenic NDV strain Texas GB, all birds in groups vaccinated with rLaSota and rLaSota/S(Y1145A) + Fct_12_ did not show any clinical signs or mortality, whereas all birds in the control group showed severe nervous signs and were euthanized at 4 dpc. These results suggest that the rNDV vectored IBV vaccines provided protection also against NDV.

The antibody titers of vaccinated birds were evaluated by the HI test. Three weeks post-immunization (prior to challenge), the means of serum HI antibody titers against NDV in groups vaccinated with rLaSota/wt.S, rLaSota/S(Y1145A) + Fct_12_ and rLaSota/SΔct + Fct_12_ reached log_2_ values of 3.2 ± 0.29, 3.0 ± 0.19 and 3.4 + 0.22 which were significantly lower than that in the rLaSota-vaccinated group (5.1 ± 0.23) (Figure 5B).

### Protective efficacy of rNDVs expressing S protein after prime-boost vaccination

**Figure 6:**
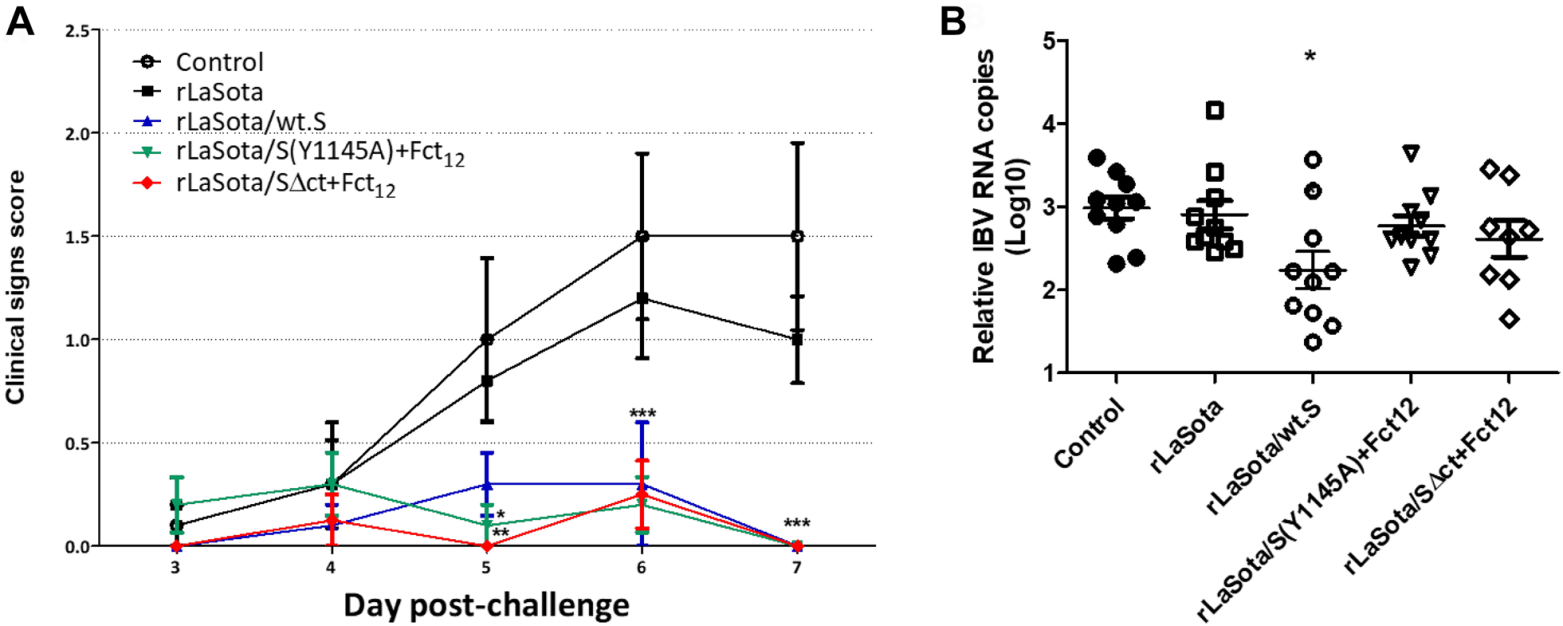
**Protective efficacy of prime-boost vaccination using rNDV-vectored IBV vaccine candidates in 1-day-old SPF chickens against IBV challenge. A** Clinical signs in chickens immunized at day 1 and day 14 of age with the indicated vaccine candidates and challenged at day 35 of age with 10^5.2^ EID_50_ per bird of Egyptian IBV variant strain EG/CU/4/2014. The birds were monitored daily for 10 days for clinical signs scoring (0 = no signs, 1 = mild ocular discharge, mild nasal discharge and/or sneezing 2 = heavy ocular discharge, heavy nasal discharge and/or coughing 3 = tracheal rales and/or mouth breathing). Mean score and SEM are plotted for each group. Statistically significant differences versus the control group at each time point are indicated by asterisk. **B** Determination of viral load (of IBV challenge virus) quantitative analysis of IBV RNA in tracheal and oropharyngeal swabs collected at 5 days post IBV challenge by RT-qPCR. Each point indicates the viral load for an individual chicken, the lines indicate the mean viral load for each group, and the error bars indicate SEM. Statistically significant differences are indicated by asterisk.

The 1-day-old SPF chicks were further evaluated for protective efficacy against challenge with the Egyptian strain IBV/Ck/EG/CU/4/2014 after prime-boost immunization with rNDVs expressing IBV S protein. After challenge with IBV/Ck/EG/CU/4/2014, birds in groups vaccinated with rLaSota/wt.S, rLaSota/S(Y1145A) + Fct_12_ and rLaSota/SΔct + Fct_12_ showed significantly lower clinical signs scores than birds in control and rLaSota-vaccinated groups at 6 and 7 dpc (*P* < 0.01) (Figure 6A). RT-qPCR results revealed significant reduction of IBV shedding in birds vaccinated with rLaSota/wt.S (*P* < 0.05) compared to the control group. Whereas, birds vaccinated with rLaSota/S(Y1145A) + Fct_12_ and rLaSota/SΔct + Fct_12_ showed very little, but not significant reduction of IBV shedding compared to the control group (Figure 6B).

**Figure 7:**
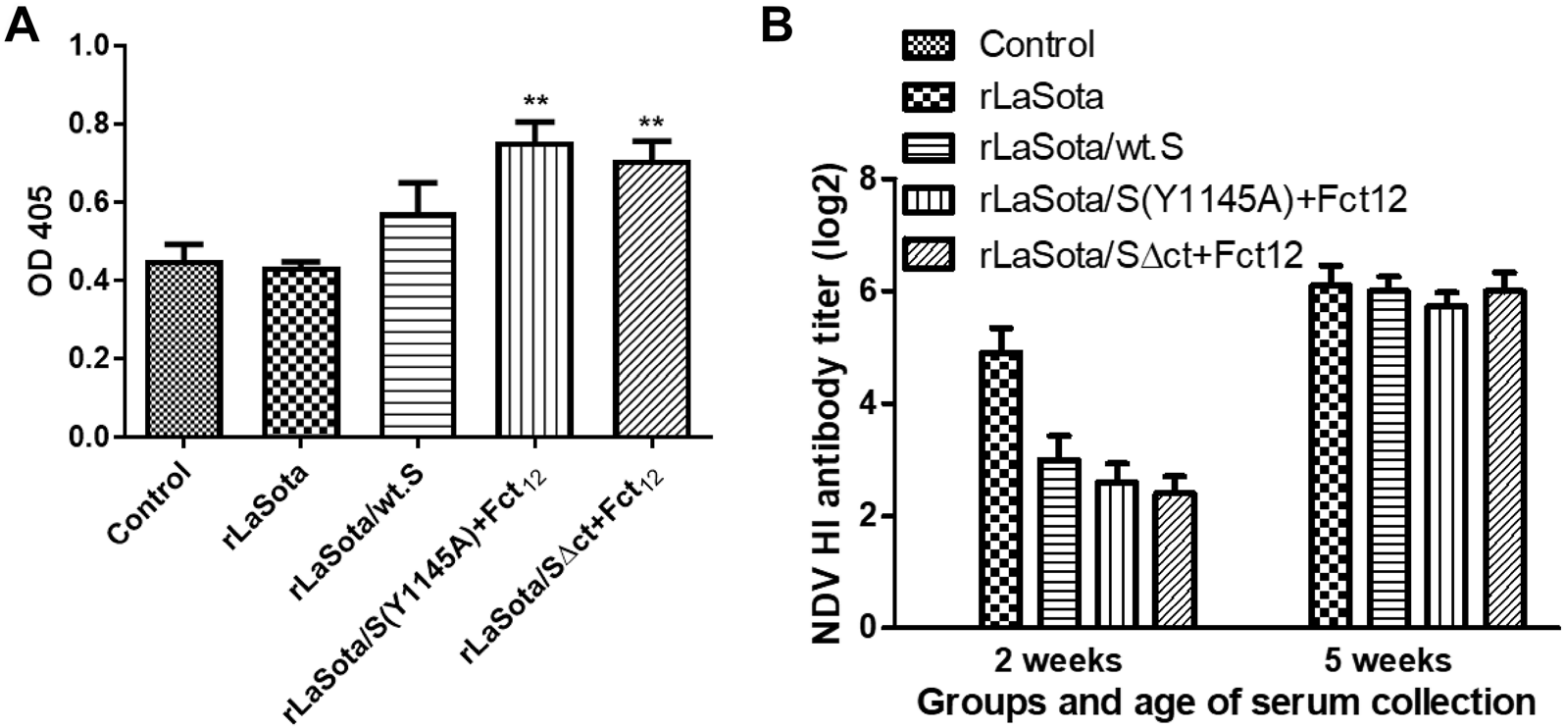
**Antibody response against IBV (A) and NDV (B) in 1-day-old SPF chicks vaccinated at day 1 and day 14 of age with different rNDV vectored IBV vaccine candidates. A** The level of IBV-specific antibodies in chicken serum samples on week 5 (prior to challenge) was determined by ELISA. Mean absorbance values and SEM are shown for each group. Statistically significant differences versus the control group are indicated by asterisk. **B** The level of NDV-specific antibody titers in chicken serum samples on week 2 (prior to boost) and on week 5 (prior to challenge) were determined by hemagglutination inhibition (HI) assay. Mean and SEM of each group are shown.

The IBV specific antibodies elicited in the vaccinated birds after prime-boost vaccination were assessed by ELISA. Three weeks after boost, chickens vaccinated with rLaSota/S(Y1145A) + Fct_12_ and rLaSota/SΔct + Fct_12_ showed significantly higher ELISA antibody titers compared to the control non-vaccinated group (*P *< 0.01). However, the chickens vaccinated with rLaSota and rLaSota/wt.S did not show IBV specific ELISA antibody titers (Figure 7A).

The antibody titers against NDV in the vaccinated birds were evaluated by HI test. Two weeks after the first vaccination (before boost), the mean serum HI titers against NDV induced by the three recombinant NDVs expressing the different forms of IBV S gene reached 2.4–3 log_2_ ± 0.31–0.42 which were significantly lower than that induced by the parental rLaSota (4.9 log_2_ ± 0.43). Three weeks after boosting (before challenge), the mean serum HI titers in these groups increased to 5.7–6 log_2_ ± 0.26–0.32 which were comparable to that of rLaSota (6.2 log_2_ ± 0.35) (Figure 7B).

## Discussion

IB is a major disease problem in the poultry industry in Egypt [17]. Currently, classic and variant live-attenuated IBV vaccines are widely used in Egypt to control IB. These vaccines have been highly successful in controlling classical IBV strains in Egypt. However, these vaccines do not provide good protection against the circulating variant strains belonging to GI-23 lineage of IBV, which is prevalent in Egypt [17, 19]. A live-attenuated vaccine belonging to GI-23 lineage may provide better protection against prevalent field strains, but development of a new live-attenuated vaccine is expensive and time consuming. Furthermore, a live-attenuated vaccine may revert to virulence, contributing to creation of new variant viruses [27]. Therefore, we have developed a vector vaccine against a prevalent Egyptian IBV variant strain. Development of a vector vaccine is rapid, and it will not lead to creation of new IBV variants.

In this study, three NDV-vectored vaccine candidates were generated using three different forms of the S protein of Egyptian IBV strain EG/CU/4/2014: rLaSota/wt.S, rLaSota/S(Y1145A) + Fct_12_ and rLaSota/SΔct + Fct_12_. The IBV S protein in rLaSota/S(Y1145A) + Fct_12_ and rLaSota/SΔct + Fct_12_ were modified to abolish the intracellular retention signal and to facilitate the transport of the IBV S protein to the cell surface [11]. This was done hypothesizing that presenting the IBV S protein at the cell surface would elicit a better humoral immune response. In addition, the modified S proteins were fused with the last 12 amino acids of NDV F protein to facilitate incorporation into the NDV virion envelope [48].

The whole S protein was used as the vaccine antigen instead of S1 or S2 protein because the S protein is highly conformation dependent. Some of the critical neutralizing epitopes on the S protein are formed by amino acids from both S1 and S2 proteins and these epitopes will be lost if they are separated. This was recently confirmed by Shirvani et al. [40]. In the present study, we have evaluated two additional forms of S protein which were not evaluated by Shirvani et al. [40].

In this study, NDV was chosen as the vaccine vector for IBV considering that the vaccine can protect against both IB and ND [40], which are enzootic in Egypt. All three NDV-vectored IBV vaccine candidates expressed S protein of IBV at high levels after five passages in ECE indicating that the S gene insertion was genetically stable. Our results showed that all three vaccine candidates grew to high titers in ECE (2^8^ HAU) which makes them suitable for mass vaccine production. All the vaccine candidates were slightly attenuated compared to the parental rLaSota virus by MDT assay.

One-day-old chickens immunized by all three rNDVs expressing the IBV S protein showed clinical protection against challenge with the Egyptian IBV strain EG/CU/4/2014. Although the three IBV vaccine candidates significantly reduced the severity of the clinical signs, they did not show reduction in tracheal viral shedding, which is another important criterion for determining the efficacy of an IBV vaccine. This result indicates that the immune responses elicited by single immunization may not be high enough to stop the virus shedding from the respiratory tract. It should be noted that IBV is a highly infectious and a fast replicating virus [49]. Therefore, it will require a high level of high affinity neutralizing antibodies and cell mediated immune response to completely block IBV replication in the respiratory tract, which is not achievable by live-attenuated IBV vaccine. Our results are in agreement with previous reports that rNDV expressing S protein prevent disease but do not stop virus shedding [40]. Another possible explanation is that the 1-day-old chicks may have immature immune system that could not have elicited a robust protective immune response after single immunization. It has been shown that vaccination of SPF chicks by IBV vaccines at day 1 of age, and to a lesser extent at day 7 of age, provided a poorly protective IBV specific immune responses, while vaccination at day 14 of age and older provided better protection against IBV challenge [50].

In the prime-boost immunization study, rLaSota/wt.S provided significant protection in terms of alleviation of clinical signs and reduction of tracheal viral shedding after IBV challenge. However, rLaSota/S(Y1145A) + Fct_12_ and rLaSota/SΔct + Fct_12_ provided only protection against the clinical disease but did not significantly decrease the tracheal viral shedding. This could be because the IBV S protein in case of rLaSota/wt.S elicited more effective cell mediated immune response, which is crucial for clearance of the IBV from the respiratory tract [1, 51–54] and also it has been reported that the humoral antibody titer does not correlate with the resistance to respiratory infection [1, 55, 56]. Another possible explanation is that the modifications done in the S protein of IBV in rLaSota/S(Y1145A) + Fct_12_ and in LaSota/SΔct + Fct_12_ could have changed the conformation of the IBV S protein and/or rendered loss of critical T cell epitopes necessary for effective blocking of IBV infection. Our results suggest that wild type S protein of IBV is the best protective antigen. Any modification to the S protein can affect the protective efficacy of the protein.

Notably, the rLaSota/wt.S provided significant protection against IBV challenge in the prime-boost vaccination experiment even though the challenge dose was increased to 10^5.2^ EID_50_ per bird. This indicates that the prime-boost vaccination strategy can provide a protective immune response against a high dose IBV challenge. There is also a need to investigate further if single-dose vaccination of chickens at older age (2-week and older) may be enough to provide a protective immune response against IBV challenge due to the well-developed immune system at this age [50].

The ELISA results showed that rLaSota/wt.S did not produce significant increase in the IBV-specific serum antibody titers. On the other hand, the rLaSota/S(Y1145A) + Fct_12_ and rLaSota/SΔct + Fct_12_ showed a significant increase in the IBV-specific antibody titers. A possible explanation is that the surface expression of the IBV S protein and its incorporation into the NDV virion, in case of rLaSota/S(Y1145A) + Fct_12_ and rLaSota/SΔct + Fct_12_ elicited much stronger humoral immune response compared to the rLaSota/wt.S which showed only intracellular expression of the S protein. Moreover, coating the ELISA plates with an inactivated whole IBV could have also increased the detection threshold of the IBV-specific humoral antibodies which were raised against S protein alone. Similarly, Eldemery and coworkers reported a higher level of antibodies against IBV S protein when S-ectodomain-coated-ELISA plates were used compared to whole-virus-coated-plates [57].

In summary, recombinant NDV strain LaSota expressing three different forms of the chicken-codon-optimized S protein of a prevalent Egyptian IBV variant strain were generated and evaluated for use as bivalent vaccines in Egypt. This study confirmed and extended the work of Shirvani et al. [40] that the whole S protein is the antigen of choice for generating an efficient vectored IBV vaccine. Our results showed that any modification to the S protein can affect its protective efficacy. Our results further showed that recombinant NDV expressing the wild type S protein of an Egyptian variant strain (rLaSota/wt.S) provided protection from clinical disease against a prevalent Egyptian variant strain of IBV and reduced virus shedding (prime-boost immunization), indicating that this vaccine candidate has the potential for controlling IBV in Egypt. Furthermore, NDV-vectored IBV vaccine will protect both IBV and NDV. This vaccine will also benefit countries neighboring to Egypt, where antigenically similar IBV strains are prevalent. Thus, suggesting that similar NDV-vectored vaccines can be engineered for use against circulating IBV strains in other geographical regions.

## Additional file

**Additional file 1.** **Cloning of three different forms of IBV S protein into pUC57.** (A) Chicken-codon-optimized wild type IBV S gene cloned into pUC57 (pUC57-CO.S). IBV S protein domains: ectodomain, transmembrane (TM) and cytoplasmic tail are represented in blue boxes, preceded by NDV gene-end (GE, in Red), intergenic (IGS, in black), gene-start (GS, in green) and Kozak (in white) sequences, and flanked by *PmeI* sites. Dotted numbers indicate amino acid positions in the cytoplasmic tail of IBV S protein. Arrows with circled numbers indicate the primers used to induce the S protein modifications using pUC57-CO.S as template (refer Table 1 for the primers). (B) Primers 1, 2, 3 and 4 used to induce the “Y1145A” mutation and add the last 12 aa of the cytoplasmic tail of NDV F protein (Fct_12_, represented in grey box) to create pUC57-CO.S(Y1145A)+Fct_12_ (C) Primers 1 and 5 used to replace the cytoplasmic tail of IBV S protein by the last 12 aa of the cytoplasmic tail of NDV F protein to create pUC57-CO.SΔct+Fct_12_.

## Abbreviations

ABSL-3+: enhanced animal biosafety level 3
CLD_50_: chicken lethal dose 50%
DAPI: 4′,6-diamidino-2-phenylindole
DMEM: Dulbecco’s minimal essential medium
dpc: days post-challenge
ECE: embryonated chicken eggs
EID_50_: egg infective dose 50%
ELISA: enzyme linked immunosorbent assay
FBS: fetal bovine serum
FITC: fluorescein isothiocyanate
HAU: hemagglutination unit
HEp-2: human epidermoid carcinoma cells
HI: hemagglutination inhibition
hpi: hours post-infection
IB: infectious bronchitis
IBV: infectious bronchitis virus
MOI: multiplicity of infection
NDV: newcastle disease virus
PFU: plaque forming unit
RBD: receptor binding domain
RIPA: radioimmunoprecipitation assay
RT-PCR: reverse transcriptase-polymerase chain reaction
RT-qPCR: reverse transcriptase-quantitative polymerase chain reaction
SDS-PAGE: sodium dodecyl sulphate–polyacrylamide gel electrophoresis
SEM: standard error of mean
SPF: specific pathogen free
TCID_50_: tissue culture infective dose 50%
TSAP: thermostable shrimp alkaline phosphatase
