# Supplementary figures and images for: Development of a recombinant Newcastle disease virus-vectored vaccine for infectious bronchitis virus variant strains circulating in Egypt
Source: Vet Res. 2019 Feb 11;50:12. doi: 10.1186/s13567-019-0631-5 (PMC6371441; doi:10.1186/s13567-019-0631-5)

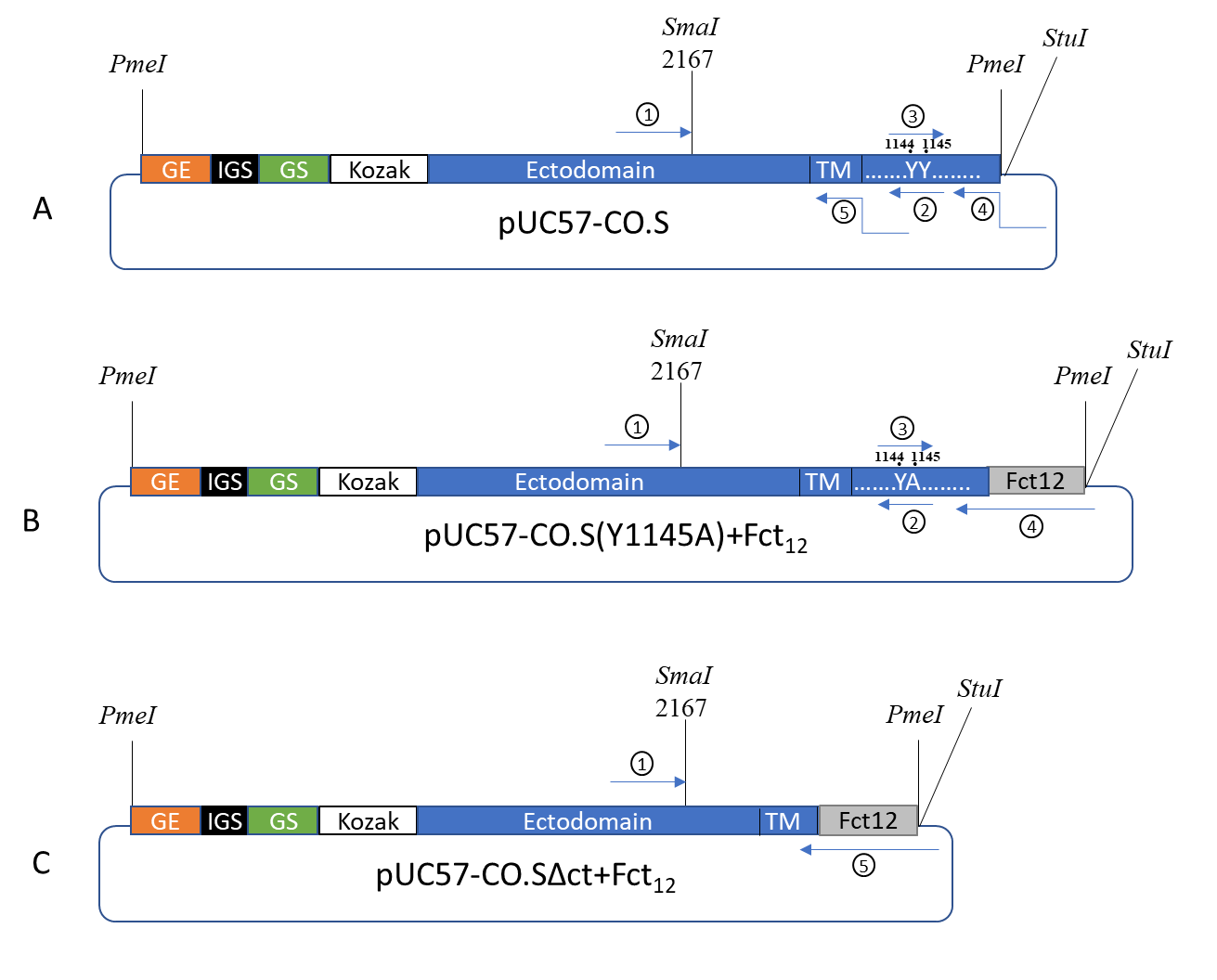

Supplement: Supplementary file 1 — Additional file 1. Cloning of three different forms of IBV S protein into pUC57. (A) Chicken-codon-optimized wild type IBV S gene cloned into pUC57 (pUC57-CO.S). IBV S protein domains: ectodomain, transmembrane (TM) and cytoplasmic tail are represented in blue boxes, preceded by NDV gene-end (GE, in Red), intergenic (IGS, in black), gene-start (GS, in green) and Kozak (in white) sequences, and flanked by PmeI sites. Dotted numbers indicate amino acid positions in the cytoplasmic tail of IBV S protein. Arrows with circled numbers indicate the primers used to induce the S protein modifications using pUC57-CO.S as template (refer Table 1 for the primers). (B) Primers 1, 2, 3 and 4 used to induce the “Y1145A” mutation and add the last 12 aa of the cytoplasmic tail of NDV F protein (Fct12, represented in grey box) to create pUC57-CO.S(Y1145A)+Fct12 (C) Primers 1 and 5 used to replace the cytoplasmic tail of IBV S protein by the last 12 aa of the cytoplasmic tail of NDV F protein to create pUC57-CO.SΔct+Fct12. [file 13567_2019_631_MOESM1_ESM.docx]
